# Development of a numerical modelling method to predict the seismic signals generated by wind farms

**DOI:** 10.1038/s41598-022-19799-w

**Published:** 2022-09-15

**Authors:** Fabian Limberger, Georg Rümpker, Michael Lindenfeld, Hagen Deckert

**Affiliations:** 1 Institute of Geosciences, Goethe-University Frankfurt, 60438 Frankfurt am Main, Germany; 2 Institute for Geothermal Resource Management (IGEM), 55411 Bingen, Germany; 3 Frankfurt Institute for Advanced Studies (FIAS), 60438 Frankfurt am Main, Germany

**Keywords:** Seismology, Geophysics, Environmental impact

## Abstract

In efforts to reduce greenhouse gas emissions, renewable energies have been increasingly leveraged to generate power; in particular, the number of wind turbines has risen sharply in recent years and continues to grow. However, being mechanically coupled to the earth, wind turbines also generate ground vibrations, which can have adverse effects on the capability of seismic observatories to detect and analyse earthquakes; nevertheless, the distances at which these signals modulate seismic records are disputed between the operators of wind farms and seismic observatories. Here, to quantify the noise signal amplitudes at distant seismometers, we develop the first numerical model to predict the seismic wavefield emitted by wind farms and simulate the complex effects of wavefield interferences, surface topography and attenuation. This modelling approach can reliably quantify the influences of multiple wind turbines on ground motion recordings and thus provide necessary information to aid decision-making in advance of wind farm installation.

## Introduction

The development and exploitation of renewable energies play a key role in slowing the warming of the global climate. Wind is a crucial source of renewable energy; consequently, increasing the number of wind turbines (WTs) to be installed in the coming decade is an important step toward a fossil-free energy supply. Nevertheless, WTs can have impacts on their environment such as audible acoustic noise, infrasound, and shadow cast. Thus, to minimize negative effects on the surrounding environment, WTs are often erected in remote areas with preferably windy conditions. Seismic stations are typically sited in similarly quiet areas to minimize the noises resulting from industries, railways, and traffic. However, research has shown that seismic stations record seismic signals produced by nearby WTs. These signals, which are considered noise, can have a significant adverse impact on the recordings of earthquakes required by various agencies to detect and analyse seismic activity. An increase in the background seismic noise at a seismic station decreases its ability to detect seismic waves emitted by earthquakes, especially if they have a small amplitude and share a common frequency range with noise signals from WTs. Therefore, governmental agencies in Germany have proposed regulations defining protected areas and minimum radii (e.g., 5 km at the Gräfenberg array in Germany^1^) that must be maintained between planned WTs and existing seismic stations. Such policies have led to strong conflicts of interest, as the operators of both WTs and seismic stations in some regions must compete for space in the same suitable areas.

With the aim of better understand the seismic emission of WTs, a number of recent studies have sought to detect and characterize the seismic noise produced by both individual and groups of WTs^1–4^. Furthermore, the correlation between meteorological data, the operation of the WT and its seismic emissions have been studied by various authors^5–7^. For instance, the interference of the wavefields emitted by multiple WTs has been modelled analytically^8^, and numerical simulations have been successfully applied to earthquakes^9–12^ and seismology^13^. Nonetheless, reliable estimates of the seismic wavefields produced by WTs and wind farms (WFs) in advance of their installation are still rather limited, making it difficult to judge whether the quality of records from nearby seismic stations might be strongly influenced by those wavefields. In particular, few approaches have been developed to model the seismic wavefields radiating from WTs, and these methods focus mostly on modelling the ground vibrations emitted by a single WT^14,15^. Additionally, although the effects of topography on earthquake waves have been studied for many years^16,17^, the effects of topography on the seismic surface waves, which are mainly produced by a WT, have yet to be elucidated.

In view of the above research gap, this study aims to develop a numerical approach to simulate the complete 3D seismic wavefield generated by 7 WTs comprising the Weilrod WF, situated northwest of Frankfurt am Main (Germany). Considering the interference of the seismic signals generated by all 7 WTs and including complex topographic effects on signal amplitudes, we compare the modelling results with observations from the closest permanent seismic station at the Taunus Observatory (TNS) located 11 km from the WF. Finally, by including wave-attenuation effects between the WF and the TNS observatory, we are able to precisely predict the noise signal amplitude at TNS based on near-field measurements. This novel approach is suitable to estimate the seismic noise field produced by WFs and to predict the noise amplitude at distant seismic stations by including effects of wavefield interferences, topography and attenuation.

## Results

### Observation of seismic signals emitted by wind turbines in the near and far fields

**Figure 1:**
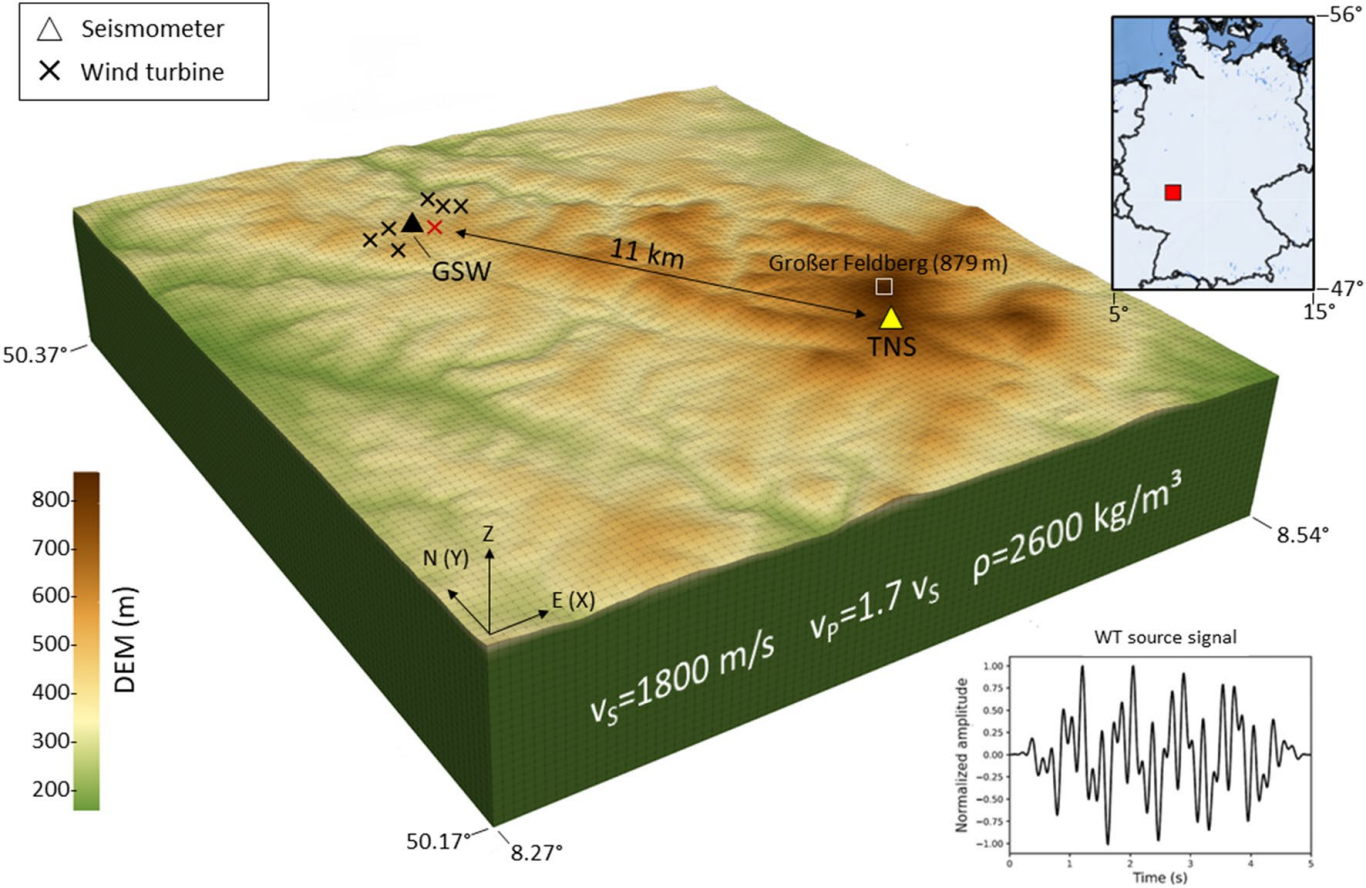
Model setup with a digital elevation model (DEM) of the local topography. The source time function of the WT used for modelling (bottom right) is obtained by summing three sinusoidal curves with frequencies of 1.15 Hz, 3.5 Hz and 6.0 Hz, each with a duration of 5 s. The temporary seismic station (GSW) is located within the Weilrod WF. The permanent seismic station (TNS) is located approximately 11 km from the WF atop the Kleiner Feldberg (about 1.3 km to the southwest of Grosser Feldberg). The coordinates of the model are given in WGS84. The thin grey lines indicate the computational mesh elements. The position of the WT used for simulations with one source only is marked by red cross. The computational model covers an area of 17 km × 19 km and a depth of 3.2 km.

Seismic station TNS is located atop the Kleiner Feldberg (825 m a.s.l.), the second largest peak northwest of Frankfurt am Main, Germany (Fig. 1). Due to its remote location far from highways, industrial areas and railways, the station has a very low noise level of < 10 nm s^−1^ and has been providing high-quality data for the permanent monitoring of earthquake activity by the German Regional Seismic Network for more than 3 decades. The Weilrod WF, situated 11 km northwest of station TNS, was erected in 2014 and currently consists of seven Nordex N117 WTs that begin to operate if the wind speed exceeds approximately 3 m s^−1^. To measure the frequency and amplitude of the near-field seismic signals emitted by these WTs, an additional temporary seismic station, GSW, was deployed from August 2015 to November 2015 in the centre of the WF. The Taunus mountain range rises to elevations between 200 and 879 m a.s.l. (the Großer Feldberg being the highest peak). The elevation along a line between GSW at the centre of the WF (source) and TNS (receiver) increases from approximately 450 m to 825 m a.s.l. but fluctuates due to several small valleys.

**Figure 2:**
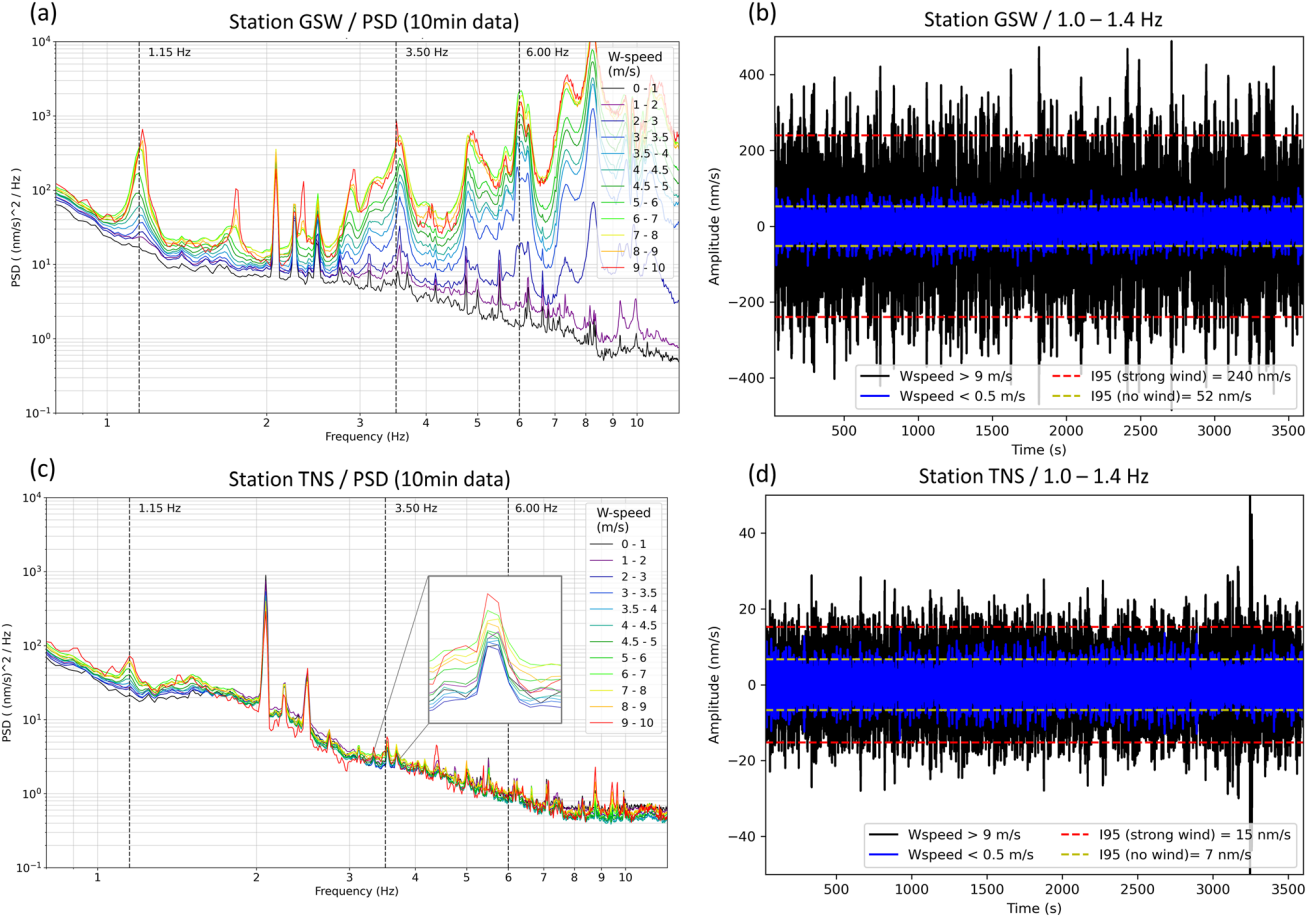
Power spectral density measured at near-field station GSW (**a**) and far-field station TNS (**c**) and correlated with the wind speed (from low wind speeds (black line) to high wind speeds (red line)) registered at the weather station Kleiner Feldberg. The amplitudes of the distinct peaks at 1.15 Hz and 3.5 Hz (inset in **c**) increase with wind speed and are observable at both stations. At TNS, the amplitudes are lower than at GSW due to amplitude decay with distance. One-hour seismological records during wind speeds > 9 m s^−1^ (black waveform) and < 0.5 m s^−1^ (blue waveform) are measured at the station GSW (**b**) and TNS (**d**). The I95 value is calculated for the four records to derive noise amplitudes at TNS and at GSW during strong and low wind conditions. It should be noted that the scale of the y-axis in (b) is an order of magnitude greater than in (**d**).

First, after processing the data (see Methods), we investigate the power spectral density (PSD) to quantify the amplitude of the seismic signals produced by the Weilrod WF at TNS and GSW. The power spectra of the data from both GSW and TNS exhibit peaks at 1.15 Hz and 3.5 Hz, which are correlated with the wind speed (Fig. 2a and c). The peak amplitude is increasing with wind speed, which agrees with the report of a recent publication showing that 1.15 Hz and 3.5 Hz spectral noise peaks are typically associated with the operation of Nordex N117 WTs^8^. However, although the WT-generated signals in the near field (at GSW) are observable between 1 and 10 Hz and even beyond 10 Hz (Fig. 2a), the amplitudes of the three sharp peaks between 2 and 3 Hz at both stations are not increasing with the wind speed, which indicates that their origin is not related to the WTs. Moreover, additional discrete high-frequency (e.g., 6.0 Hz) peaks are observed at the near-field station (GSW), whose data are dominated by the WT-emitted signals (GSW is only 150 m from the closest WT), whereas this peak is not identified at TNS. These observations support the assumption that the two spectral peaks at 1.15 Hz and 3.5 Hz observed at TNS are associated with the operation of the Weilrod WF but are reduced in amplitude relative to those at GSW due to the decay of wave energy with increasing distance from the WF. Signals beyond 3.5 Hz that are detectable at GSW are not observable at TNS, due to attenuation and geometrical spreading effects and therefore are not considered further in the modelling.

Next, to better quantify the spectral noise amplitudes of the WF signals with 1.15 Hz in terms of maximum ground velocities at GSW and TNS (Fig. 2b and d), we determine the I95^18^ values (95.45% of the amplitudes are within two times the standard deviation) of the bandpass-filtered signals with cutoff frequencies of 1 Hz and 1.4 Hz. As in similar previous studies, we use noise levels in units of ground motion velocity to allow for a more direct comparison with signal amplitudes. However, corresponding values for acceleration and displacement at station GSW and TNS are listed in the supplements (Tab. S1). For each station, we select a 1-h time segment with a wind speed < 0.5 m s^−1^ and another one-hour time segment with a wind speed > 9 m s^−1^. The I95 noise amplitudes at GSW (TNS) in the near (far) field are approximately 240 nm s^−1^ (15 nm s^−1^) at a high wind speed and 52 nm s^−1^ (7 nm s^−1^) at a low wind speed. We conclude that at high wind speeds and within the frequency range of 1–1.4 Hz, the Weilrod WF, on average, causes a 2.1-fold increase (from 7 to 15 nm s^−1^) in the noise level at TNS and that the seismic signals produced by the Weilrod WF are detectable in 11 km at TNS, because of the overall low noise level at TNS. Such an increase can affect the ability to detect small earthquakes and it can also have an impact on the determination of earthquake magnitude.

### Model setup for simulating the seismic signals from WTs

The numerical simulations are based on a 3D model (17 km × 19 km) of the uppermost crust (depth of 3.2 km) and surface topography in the region around the Weilrod WF and station TNS (Fig. 1). The model is a homogeneous half-space characterized by isotropic physical properties (no geological layering). We assign a uniform shear-wave velocity of $v_{S} = 1800\;\;{\text{ m}}\;{\text{ s}}^{ - 1}$ and compressional-wave velocity of

1 $$ v_{P} = 1.7 v_{S} $$

and the density is set to 2600 kg m^−3^. In a first step, anelastic absorption (attenuation) of waves is not included in the model to focus on topographic effects. However, further below we will include attenuation effects by specification of the seismic quality factors Q_S_ (for S-wave propagation) and Q_P_ (for P-wave propagation). The location of the source is set on the surface at the coordinate of one of the seven WTs in Weilrod (WT4 in Table 1). The source features only a vertical component (Z) to simulate an up- and downward motion at the foundation of the WT. A receiver is located at the location of TNS to extract synthetic waveforms during the numerical forward modelling. For the source time function, we sum three sinusoidal functions with frequencies of 1.15 Hz, 3.50 Hz and 6 Hz (each with a duration of 5 s), which correspond to the characteristic frequencies of the three peaks measured at GSW and TNS (Fig. 2a and c). The topography in the model is defined based on a digital elevation model (DEM) using data with a resolution of 30 m from the Global Multi-Resolution Topography (GMRT) synthesis project^19^.

**Table 1:** Coordinates of seismic station TNS and GSW as well as of the WTs in UTM 32U projection.

	Longitude (UTM 32U)	Latitude (UTM 32U)
TNS	460513	5563438
GSW	454005	5572704
WT1	453863	5572310
WT2	453873	5572779
WT3	453421	5572819
WT4*	454786	5572972
WT5	455338	5573613
WT6	454889	5573642
WT7	454789	5574054

WT4* is used as the source for the modelling of the radiation from a single WT.

### Simulating synthetic waveforms at TNS

To study the effects of topography on the signal amplitude at TNS, we perform simulations in an isotropic half-space model (see Methods) both including and excluding topography. To simulate wave propagation, we use the software package Salvus^20^ provided by Mondaic AG/Ltd in Zurich, Switzerland. The Z, N (Y) and E (X) components of each synthetic seismogram at TNS are bandpass filtered in three frequency bands: 1.15 ± 0.3 Hz, 3.5 ± 0.3 Hz and 6.0 ± 0.3 Hz. By comparing the two model simulations with and without topography, we find that including topography reduces the signal amplitudes on all components of the 6.0 Hz signal and the Z and E components of the 3.5 Hz signal (see supplements Fig. S1), which can be explained by the scattering and reflection of waves along their paths. In contrast, the amplitudes on the N component of the 3.5 Hz signal and on all three components of the 1.15 Hz signal are greater with topography than they are without topography (see supplements Fig. S1), indicating that topography has an amplifying effect on WT-emitted signals at comparatively low frequencies. While high-frequency waves particularly suffer from scattering due to topography, low-frequency waves seem to be focused and modulated in a constructive manner along their travel path; this amplifying effect is observed in a similar way concerning earthquake waves^16,17^.

### Radiation from a single wind turbine

**Figure 3:**
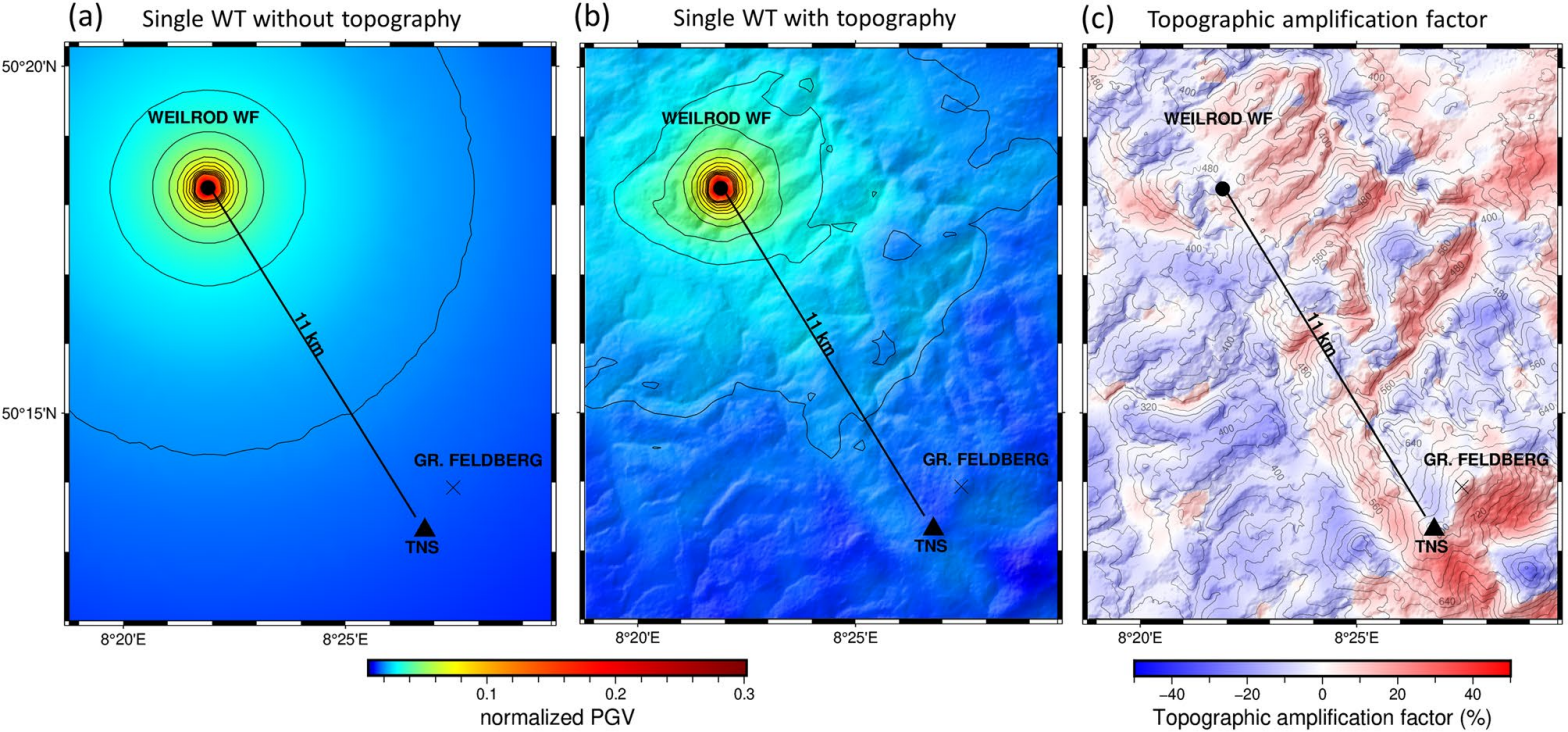
Peak ground velocity (PGV) maps without (**a**) and with (**b**) topography and the resulting map of the amplification due to topography (**c**) for a single WT at the centre of the Weilrod WF showing that the amplitude increases at the permanent station (TNS). The amplitudes in (**a**) and (**b**) are normalized to the maximum amplitude in Fig. 4c (with 7 WTs), to compare the radiation of a single and multiple WTs. Note: The contour lines in (**c**) are representative of the digital elevation model.

Here, to further investigate the spatially varying effects of topography on low-frequency signals, we simulate and analyse the propagation of surface waves at 1.15 Hz (the dominant frequency of the signals emitted by the WTs) using a 1.15 Hz sinusoidal source time function and generate maps of the vertical peak ground velocity (PGV) both with and without topography (Fig. 3a and b). In both cases, we use a single WT at the centre of the Weilrod WF as the source. For both model setups, we plot the spatial distribution of the topographic amplification factor *A* (Fig. 3c) by calculating the signal amplification or reduction in percent (%) based on Eq. (2)^16^:

2 $$ A = \left( {\frac{{PGV_{w} }}{{PGV_{wo} }} - 1} \right) \times 100 $$

where PGV_w_ denotes the PGV obtained with topography and PGV_wo_ denotes the PGV obtained without topography. The map of the resulting amplification factor *A* (Fig. 3c) indicates that the amplitudes of 1.15 Hz signals are significantly modulated by topography. This effect is especially pronounced on the mountainside to the south–southeast of TNS and Großer Feldberg, reflecting the apparent correlation between the reduction and amplification of the PGV with the DEM. In contrast, the mountain ridge between the WT source and TNS appears to act as a wave guide and preserves the signal amplitude along its path, thus opposing the expected reduction with geometrical spreading. Generally, however, the PGVs decrease with increasing distance from the WF. Even for a single WT in Weilrod, however, we can demonstrate the amplifying effect of the topography on the signal amplitude near TNS.

**Figure 4:**
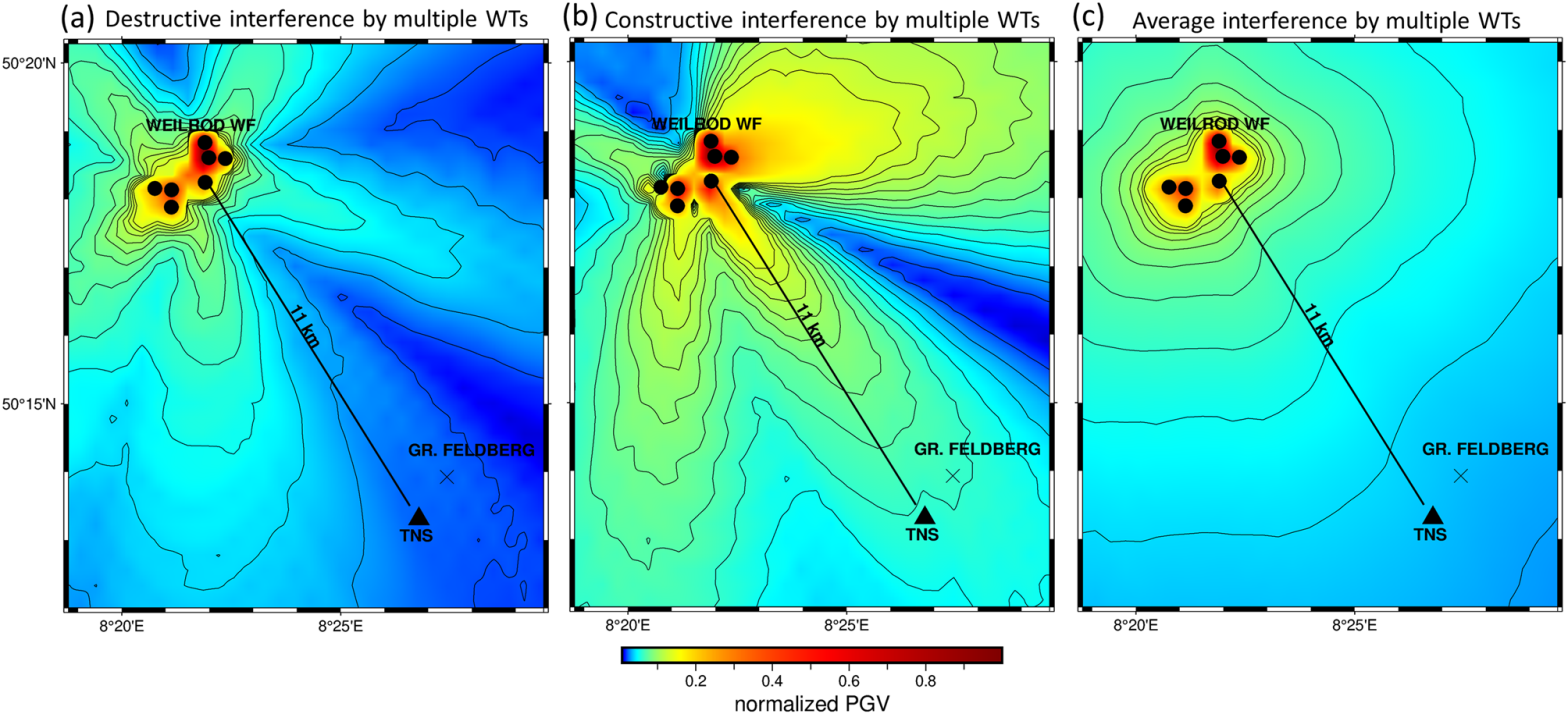
The specific destructive (**a**) and constructive (**b**) interference of the wavefields produced by multiple WTs results in low and high normalized PGVs, respectively, at TNS. The interference (radiation) pattern in (**c**) is obtained by averaging 100 PGV maps calculated with a randomly chosen phase for each source time function of the 7 sources. Topography is not included here. The amplitudes are normalized to the maximum amplitude that occurs and is therefore 1 at the source location.

### Radiation from multiple wind turbines considering the effects of wavefield interference

The wavefield of a single WT can differ significantly from the complete wavefield of an entire WF due to constructive and destructive interference^8^. To consider these effects in detail, we expand our study and place a source at each of the 7 WTs in the Weilrod WF and numerically simulate 100 PGV maps without topography using a randomly chosen signal phase of the sinusoidal time function for each of the seven sources. The modelling results show both destructive interferences, resulting in a low PGV at TNS (Fig. 4a), and constructive interference, yielding relatively high PGVs (Fig. 4b). Since the WTs are not all expected to vibrate in phase, a single interference pattern can represent only a snapshot of the ground motion before the radiation generates another pattern. Therefore, to derive a representative radiation pattern, we averaged 100 different PGV maps (Fig. 4c), thereby avoiding the predominance of any single interference pattern. The resulting average PGV map (Fig. 4c) shows the decrease in amplitude with increasing distance from the WF, but the obtained pattern differs clearly from the two patterns with either only destructive interference or only constructive interference.

### Radiation from multiple wind turbines considering the effects of wavefield interference, topography, and attenuation

**Figure 5:**
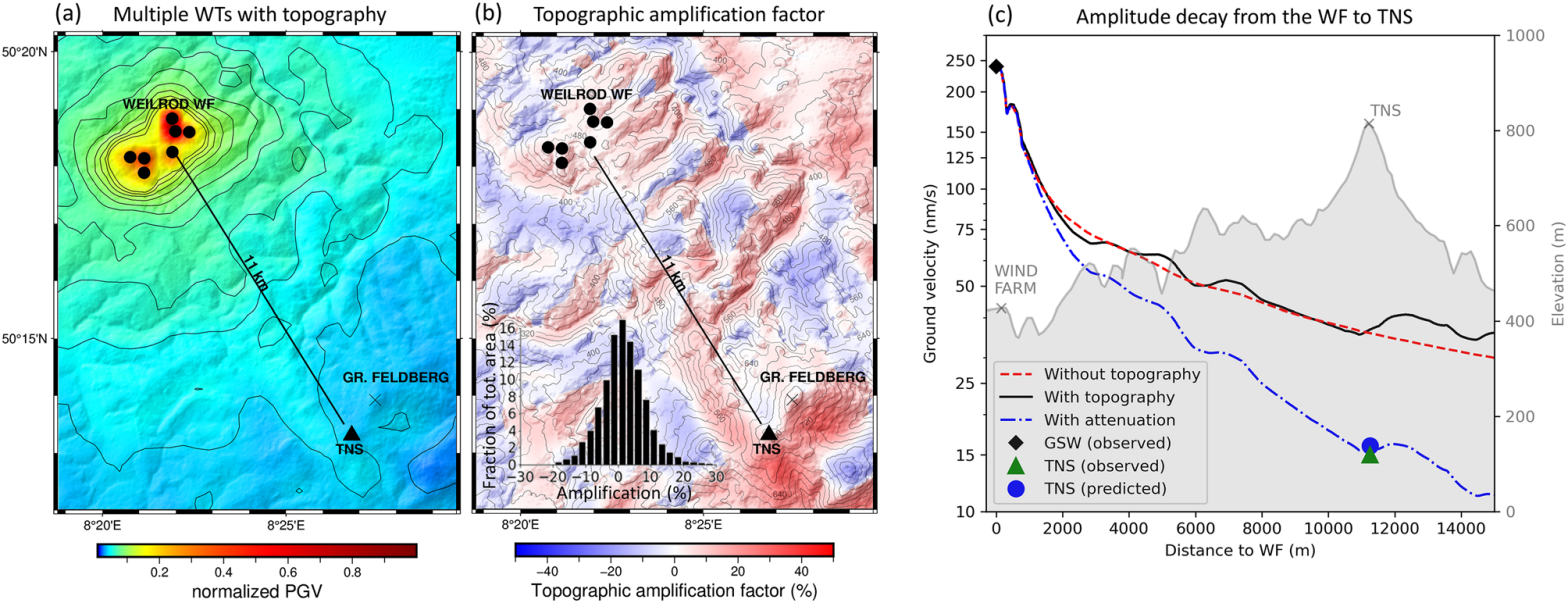
Maps of the average peak ground velocity (PGV) with topography (**a**) and the amplification factor due to topography (**b**) for all seven WTs in the Weilrod WF. On average, the amplification at TNS due to topography is about 9%. The PGVs along a straight line between the centre of the WF (station GSW) and permanent station (TNS) are extracted and calibrated with respect to the measured PGVs at the temporary station (GSW) within the WF (**c**). The measured amplitude at TNS is finally predicted including attenuation in the model. The distribution of the obtained amplification factor in the model domain ranges from − 20 to 30% (histogram in **b**). The amplitudes in (**a**) are normalized to the maximum amplitude that occurs and is therefore 1 at the sources. Note: The contour lines in (**b**) are based on the digital elevation model and the left y-axis in (**c**) is logarithmic.

Finally, we calculate the average PGV distribution produced by the whole Weilrod WF considering both the effects of topography and the interference caused by the emissions of multiple WTs (Fig. 5a). The same set of randomly chosen phases of the source time functions used for the case without topography (Fig. 4c) is used again in this case, and 100 individual PGV maps are averaged, allowing us to compare the average PGV distributions obtained without (Fig. 4c) and with (Fig. 5b) topography. In the same manner as Fig. 3c, a map of the PGV amplification factor is obtained (Fig. 5b), the distribution of which reveals that amplitudes are preserved along the mountain ridge between the WF and TNS if topography is included in the model. The minimum amplification factor in the study area is − 20% (an amplitude reduction of 20%), while the maximum value is 30% (inset in Fig. 5b); however, such high amplification factors (values > 20%) are limited to a small proportion (< 1%) of the total area. Generally, the map of the amplification factor is comparable to that in the scenario with only a single WT (Fig. 4c), although considering all seven WTs causes some amplification areas to be enlarged, generally along the mountainsides facing away from the source (e.g., to the south of TNS and Großer Feldberg, similar to Fig. 4c). In contrast, amplitude reductions are associated mostly with valleys^16^. By comparing the synthetic waveforms at TNS for each of the 100 interference scenarios with and without topography, we infer that the amplitude at TNS increases by approximately 9% on average if topography is considered (see supplements Fig. S2). Generally, with respect to the 100 specific interferences, an amplification due to the topography near TNS is much more likely than a reduction (see supplements Fig. S2).

To further investigate the decay of the PGV, we extract the PGVs along a straight line connecting the location of GSW to TNS and plot the simulation results both including and excluding topography (Fig. 5c). The amplitude steadily decreases logarithmically with increasing distance from the WF if topography is not included, as expected, whereas the amplitude decreases globally with topography but increases locally (e.g., at distances of 4–5 km, 6–8 km and 11–15 km). The sudden increase of amplitudes at a distance of 500 m is observable for the case with and without topography and is likely a consequence of wavefield interferences. In case of attenuation using Q_S_ = 25 and Q_P_ = 40, the amplitude decay with distance is higher; however, the local topographic effects remain. As mentioned before, the amplitudes significantly increase on the mountainside behind TNS opposite the WF. The PGVs along the line are calibrated to the noise amplitude (I95) of approximately 240 nm s^−1^ measured at GSW for wind speeds > 9 m s^−1^ (Fig. 2b). This means that the simulated amplitude at the location of GSW is matched up with the I95 value measured at GSW. Furthermore, we measure an amplitude (I95) of approximately 15 nm s^−1^ at TNS at wind speeds > 9 m s^−1^ (Fig. 2d). The resulting simulated amplitude including the effects of interferences, topography, and attenuation fits well with the observed amplitude of 15 nm s^−1^ at TNS, which means that the measured amplitude at TNS is predictable if these effects are included. Overall, this analysis demonstrates that the amplitude of noise at TNS (and other areas in the Taunus region) caused by low-frequency WT signals is underestimated if topography is neglected.

## Discussion

In this study, we present a novel numerical modelling approach to predict seismic noise amplitudes generated by a WF and we show that wavefield interferences of multiple WTs significantly affect the seismic radiation pattern of a WF. Furthermore, we demonstrate that topographic effects lead to local reductions, but also amplifications of the noise signal amplitude, especially for low-frequency signals. Finally, we show that we can predict the noise amplitude at a distant station using near-field measurements at a WF if attenuation is assigned to the model. In terms of aiming noise amplitude predictions, these effects can be significant which is why they should be considered in the modelling process.

Characteristic seismic signals produced by the Weilrod WF are detectable 11 km away at the TNS seismic observatory. The noise amplitudes regarding the I95 criteria are derived from both near- and far-field measurements at wind speeds above a sufficiently high threshold of 9 m s^−1^, at which the WTs are fully operating. The amplitudes show a significant increase in the seismic noise level at a frequency of 1.15 Hz, which is due to the operation of the WTs. Particularly the low-frequency signals emitted by WTs are of importance since the corresponding waves travel far (here 11 km) due to a weak wave damping and these signals are therefore widely detected^2,8^. Using our 3D model, we numerically simulate the seismic emissions of seven WTs at the Weilrod WF both including and excluding topography. When topography is included during the modelling, the synthetic seismograms at TNS located atop the Kleiner Feldberg display reduced signal amplitudes at high frequencies (e.g., 6.0 Hz) but amplified signals at low frequencies (1.15 Hz). Upon plotting the distribution of the modelled PGV, we find a systematic correlation between the topography and the amplification factor induced by the terrain, such as amplifying and reducing effects of mountain ridges and valleys, respectively^16^. Averaging the seismic radiation patterns guarantees that no single constructive or destructive interference pattern is dominant, and therefore, the average radiation pattern better approximates the representative seismic radiation of the WF. This demonstrates that the inclusion of interferences, topography and attenuation enables the amplitude of WT-induced noise at a distant seismometer to be accurately estimated. Such a prediction requires calibration measurements in the near field of the corresponding WF. If the predictions are necessary before the installation of a WF, we recommend to measure seismic amplitudes in the near field of WTs that have the same or similar type and dimensions as the planned WTs, to enable a sufficient calibration of the modelled amplitudes. Generally, different WT types and numbers can be considered in one and the same model. However, predictions of the signals produced by WTs in a region with pronounced terrain could underestimate the noise amplitude if the local topography is neglected.

The data analysis and modelling in this study are based on specific assumptions and the data used have some limitations to be discussed. We correlate the seismic records with data of wind speeds measured at the TNS observatory and not at the Weilrod WF. A more accurate correlation could be achieved using operational data from the WTs (e.g., rotation rate)^6^, however, this data was not available for this study. To calibrate the simulated noise amplitudes at the distant seismometer, we use measurements from one seismic station in the near field of the WF assuming that geological and topographic effects along this short distance from the WT to the seismometer are negligible. This calibration could be improved by using two or more near-field measurements to increase the reliability of the calibration. Furthermore, we assign specific attenuation values to model the noise amplitude at the distant station. The chosen quality factors are plausible to describe upper crustal damping of seismic waves^21,22^, however, different values could be considered. Finally, large-scale geological structures in the subsurface, which are not included in this study, might have a significant effect on the wave propagation as well. In view of these points, estimating the amplitudes of noisy signals emitted by a WF prior to its installation encounters three main challenges: the limited availability of robust calibration measurements at a WT with the specifications needed to calibrate the source amplitude, the lack of information regarding possible major structures and geophysical properties in the shallow subsurface, and the lack of access to reliable meteorological data or operational data of the WTs. However, missing information on the subsurface can be obtained by, e.g., using geophysical imaging. Furthermore, the lack of precise operational data of the WTs can be sufficiently filled using open access meteorological data provided by weather services, as we demonstrate in our study. To finally evaluate the effect of a WF on a nearby seismometer, the background noise level measured at seismic stations should be considered, and from this relationship, the effect of a new WF on seismometer measurements can be assessed.

Our results show that the presented modelling approach is capable of simulating the effects of topography, wavefield interference and attenuation and therefore can provide higher-accuracy predictions of the amplitudes of noisy signals generated by multiple WTs. Our approach has been validated with data measured at a temporary seismic station within an existing WF and a permanent station at a distant seismic observatory. A preliminary version of the approach was tested in a previous study^8^. As the first numerical modelling approach that includes topography, interferences and attenuation, our approach can be adapted to consider arbitrary WF geometries. Furthermore, it can be employed in different locations to predict the potential influences of planned WFs on nearby seismometers, thus providing necessary information for authorities and agencies as well as the operators of WFs and seismic observatories. Possible applications are, as presented, the estimation of the seismic radiation of a single WT, multiple WTs or WFs in a region and the effect of replacing (repowering) old WTs by modern ones. In future, the approach will be applied and extended to study the influence of geological structures, specific noise-reducing WF geometries and various source mechanisms of the WTs. This would further improve the understanding of seismic emissions from a WF and would finally result in more precise predictions.

## Methods

### Data processing and power spectral density

We analysed continuous data (100 Hz sampling frequency) recorded at the two seismic stations, TNS and GSW, between August 20, 2015, and November 26, 2015. At TNS, a Streckeisen STS-2 Sensor and a REFTEK 130 data logger are permanently installed. At GSW, a Nanometrics Trillium Compact (120 s) sensor and a Nanometrics Taurus data logger were installed. The data was restituted to derive true ground motions (ground velocity). We cut the data into 10-min time segments and obtained approximately 15,000 time segments per station, yielding 30,000 segments in total. The power spectral density (PSD) was calculated for each time segment for both data sets using the method proposed by Welch (1967)^23^. We used a moving window length of 60 s to compute the PSD for each 10-min time segment; thus, the PSD of each 10-min segment was generated by averaging the PSDs of 10 shorter segments. Then, to each of the average PSDs derived from the 30,000 10-min time segments, we assigned a wind speed based on meteorological data with a 10-min sampling interval provided by the German Weather Service (weather station Kleiner Feldberg)^24^. The data were assigned by matching up the times of the seismic and meteorological records. Using bin sizes of 0.5 m s^−1^ or 1 m s^−1^, the PSDs were then binned into wind speeds between 0 and 10 m s^−1^ because the wind speed never exceeded 10 m s^−1^ (during the time period of the measurements). To remove outliers and transient or undesired signals (related to, e.g., mining explosions or earthquakes), we clipped the PSD data set for each station by excluding the 50% of the data set corresponding to the largest spectral amplitudes because we found that the frequency peaks in the spectra became sharper by removing 50% of the data before averaging. For the remaining 50%, we calculated the average power spectrum for each wind speed bin (Fig. 2).

### Quantification of noise amplitudes

With the aim of deriving noise amplitudes as ground velocities with respect to the I95^18^ criteria at both stations in the case of high and low wind speeds, we examined the meteorological data for one-hour time windows with average wind speeds of < 0.5 m s^−1^ (2015-10-19, 03.00 a.m.) and > 9 m s^−1^ (2015-11-18, 4:00 a.m.) (Fig. 2). For these time periods, we bandpass-filtered the corresponding seismic records from GSW and TNS between 1 and 1.4 Hz with a 4th-order butterworth filter and calculated the corresponding I95 values. We are aware that we compare I95 values (from observations) with PGVs (from simulations), however, PGVs are not suitable to properly describe the observed noise amplitude of the harmonic continuous noise signals from the WTs we were dealing with at TNS and GSW. Therefore, we presumed the I95 values to be representative as a quantification of the noise level and hence to be suitable to be compared with the simulated PGVs.

### Numerical simulation of a single wind turbine

To numerically simulate the propagation of waves through an isotropic medium (i.e., ground motions) at the far-field station (TNS), we used the commercial software package Salvus^20^, which is a suite of software for performing full waveform modelling and inversion provided by Mondaic AG/Ltd. (Zurich). The topography of the model was obtained from the Global Multi-Resolution Topography (GMRT) synthesis project^19^. The model had an extension of 17 km × 19 km and a depth of 3.2 km. The simulations are performed based on a mesh with minimum 2 elements per signal wavelength. However, to guarantee accurate simulation results, we considered the source frequency (e.g., 1.15 Hz) plus 1 Hz to be the reference for the simulation. Therefore, we derived a grid spacing of about 420 m (1800 m s^−1^/2.15 Hz/2 elements) when simulating 1.15 Hz signals with an S-wave velocity of 1800 m s^−1^. This also implies that we used a coarser grid for simulation of 1.15 Hz signals than for simulations with 6 Hz signals. We tested the sensitivity of the results to the resolution using 2 and 4 elements per wavelength and we found no significant difference between the results. The meshing is done internally by the software. The model had a free surface at the top, but sufficiently absorbing boundaries at all other sides. To model the seismic source, we placed a vertical force vector of 1 N m at a WT in the centre of the Weilrod WF. The source is located at the surface of the computational domain at the position of a WT. Based on the signals measured at both GSW and TNS, we simulated the propagation of the wavefield with dominant frequencies of 1.15 Hz, 3.5 Hz and 6 Hz in a homogenous half-space both with and without topography. The source time function was a sum of a 1.15 Hz, 3.5 Hz and 6 Hz sinus function and is tapered with a Tukey window to avoid discontinuities at the beginning and end of the source wavelet.

### Numerical simulation of multiple wind turbines

To consider the effects of wavefield interference, we simulated 100 wavefields (scenarios), each with a randomly chosen set of signal phases at each of the 7 WTs (sources), using a 1.15 Hz sinus wavelet as a source time function. Instead of using a random signal phase^8^ between 0 and $\pi$, we used a value between 0 and 2$\pi$ to consider as many as possible scenarios of wave interferences. Then, we extracted the peak ground velocity (PGV), which means the maximum occurring magnitude of amplitude at each element in the mesh, for all scenarios. Finally, the PGVs of all scenarios were averaged to obtain a radiation pattern that was not dominated by a single interference source. Then, we interpolated the resulting surface data for better visualization. This procedure is represented graphically in the supplements (Fig. S3) and was executed for the cases both with and without topography. To determine the spatial distribution of the amplification due to topography, the topographic amplification factor was computed from the two average radiation patterns using Eq. (1). The simulated amplitude decay from the WF to TNS was extracted from the simulated radiation pattern and calibrated with the noise amplitude (I95) obtained from measurements at GSW. Finally, we performed the simulation of 100 wavefields including attenuation using seismic quality factors of Q_S_ = 25 (S-wave) and Q_P_ = 40 (P-wave). Again, the simulated amplitude decay was calibrated with the amplitude values obtained from measurements at GSW. The quality factors were chosen in a way, that the predicted amplitude at TNS approximately matched up with the observed amplitude.

## Supplementary Information

Supplementary Information.

## Data Availability

The raw seismological data that were processed in this study are available from Goethe University Frankfurt. The meteorological data is open source and is provided by the German Weather Service^24^. All datasets used and analysed during the current study are available from the corresponding author (limberger@igem-energie.de) on request.

## Code availability

Data processing was performed using the Python packages NumPy, SciPy and ObsPy^25^. The numerical simulations were performed using the commercial software package Salvus^20^. The Figures were partly prepared using Python Generic Mapping Tool (PyGMT v0.5.0, www.pygmt.org)^26^.
