# Supplementary material for: Development of a numerical modelling method to predict the seismic signals generated by wind farms
Source: Sci Rep. 2022 Sep 15;12:15516. doi: 10.1038/s41598-022-19799-w (PMC9478089; doi:10.1038/s41598-022-19799-w)
Supplement: Supplementary file 1 — Supplementary Information. [file 41598_2022_19799_MOESM1_ESM.docx]

Supplementary figures


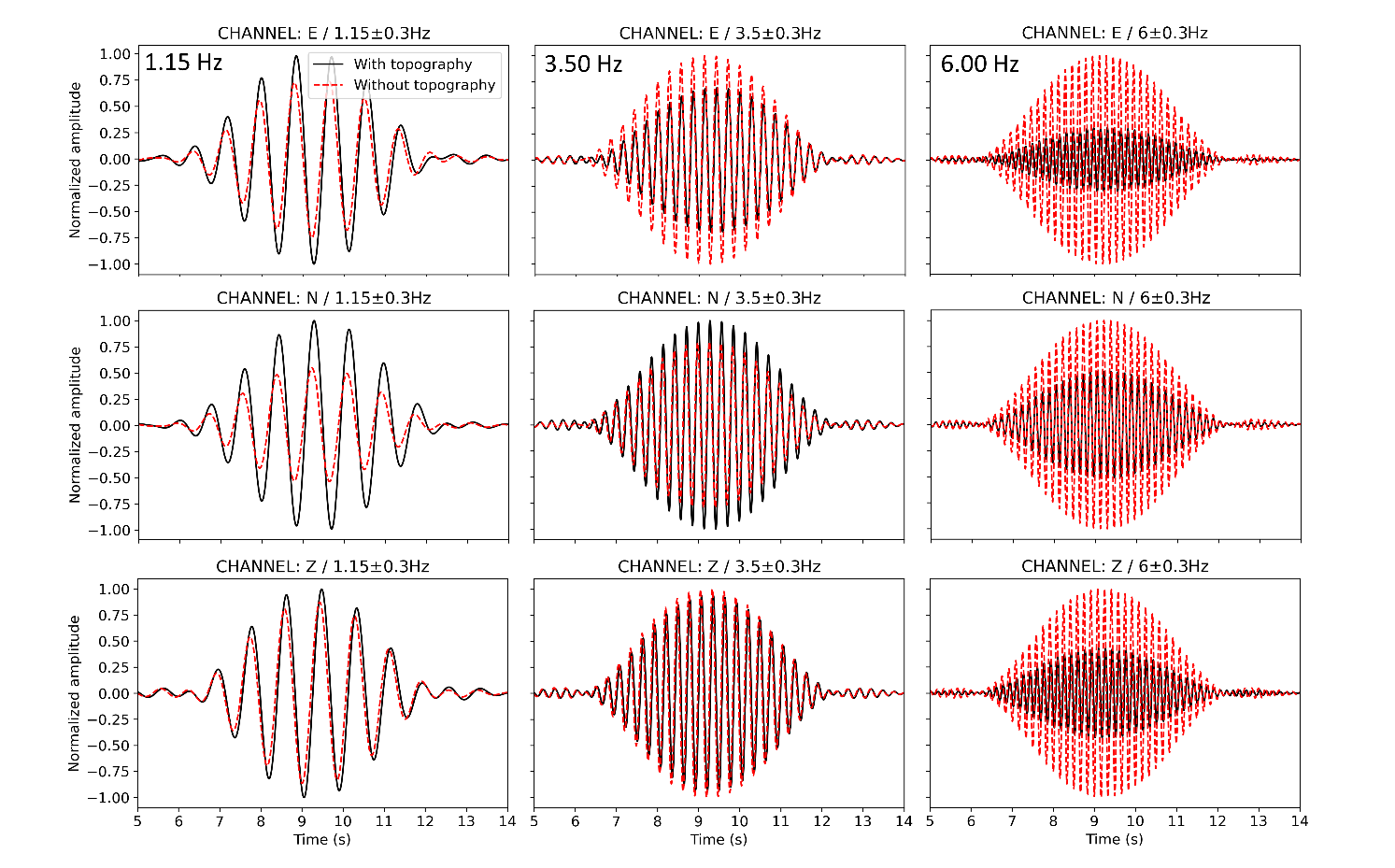


Figure S1: Synthetic waveforms of the E (top), N (middle) and Z (bottom) components simulated at the permanent seismic station (TNS) and filtered within frequency bands of 0.85–1.45 Hz (left), 3.2–3.8 Hz (middle) and 5.7–6.3 Hz (right). The low-frequency signal components are clearly amplified due to topography in comparison with the high-frequency signals.


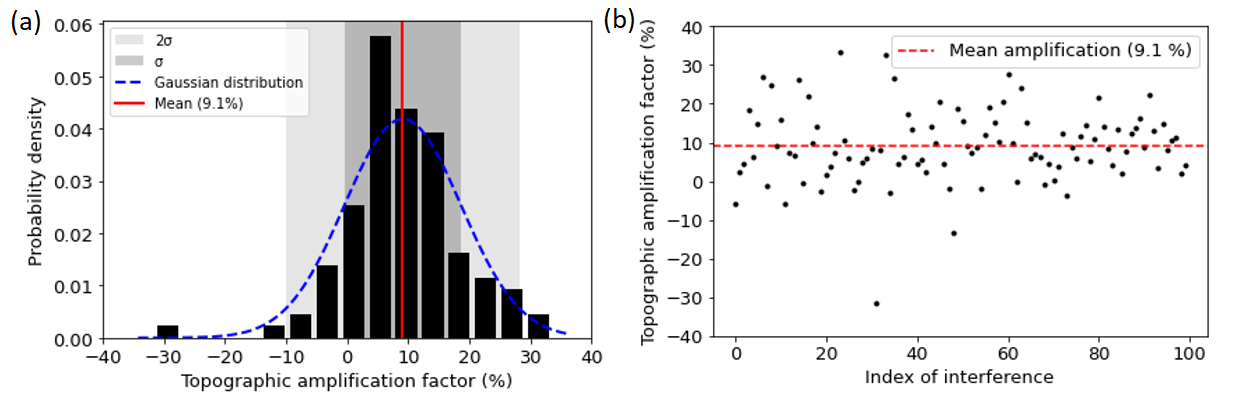


Figure S2: (a) The distribution of 1.15 Hz signal amplification at TNS due to topography for the 100 scenarios of interferences. The mean amplification is 9.1 %. The range is from -31 % to 34 %. However, -31 % is an outlier and an amplification is likelier than a reduction of amplitude (b).


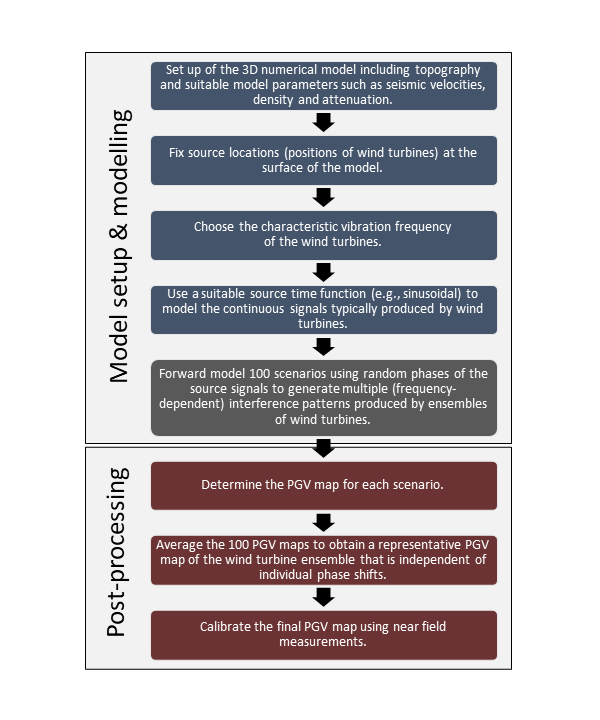


Figure S3: The flow chart represents the procedure of modelling the radiation pattern of wind turbines used in the study.

Table S1: I95 values for ground acceleration, velocity and displacement at station GSW and TNS during conditions with strong wind and no wind.

|  | Acceleration | | Velocity | | Displacement | |
| --- | --- | --- | --- | --- | --- | --- |
|  | Strong wind | No wind | Strong wind | No wind | Strong wind | No wind |
| GSW | 1734 nm/s² | 375 nm/ s² | 240 nm/s | 52 nm/s | 33 nm | 7 nm |
| TNS | 108 nm/s² | 50 nm/s² | 15 nm/s | 7 nm/s | 2 nm | 1nm |
